# Comparative Analysis of Infection by *Rickettsia rickettsii* Sheila Smith and Taiaçu Strains in a Murine Model

**DOI:** 10.3390/pathogens9090744

**Published:** 2020-09-10

**Authors:** Eliane Esteves, Chanida Fongsaran, Ingeborg M. Langohr, Sean P. Riley, Marcelo B. Labruna, Sirlei Daffre, Andréa C. Fogaça, Kevin R. Macaluso

**Affiliations:** 1 Department of Parasitology, Institute of Biomedical Sciences, University of Sao Paulo, Sao Paulo 05508-900, Brazil; sidaffre@icb.usp.br (S.D.); deafog@usp.br (A.C.F.); 2 Department of Pathobiological Sciences, School of Veterinary Medicine, Louisiana State University, Baton Rouge, LA 70803, USA; chanida@lsu.edu (C.F.); ilangohr@lsu.edu (I.M.L.); sriley3@umd.edu (S.P.R.); 3 Department of Veterinary Medicine, University of Maryland-College Park and Virginia-Maryland College of Veterinary Medicine, College Park, MD 20742, USA; 4 Department of Preventive Veterinary Medicine and Animal Health, School of Veterinary Medicine and Animal Science, University of Sao Paulo, Sao Paulo 05508-270, Brazil; labruna@usp.br; 5 Department of Microbiology and Immunology, College of Medicine, University of South Alabama, Mobile, AL 36688, USA

**Keywords:** *Rickettsia rickettsii*, Taiaçu strain, Sheila Smith strain, Rocky Mountain spotted fever, Virulence, C3H/HeN mice

## Abstract

Rocky Mountain spotted fever (RMSF) is a life-threatening tick-borne disease caused by *Rickettsia rickettsii*, which is widely distributed throughout the Americas. Over 4000 cases of RMSF are recorded annually in the United States, while only around 100 cases are reported in Brazil. Conversely, while case fatality rates in the United States oscillate around 5%, in Brazil they can surpass 70%, suggesting that differences in tick vectoring capacity, population sensitivity, and/or variability in virulence of the rickettsial strains may exist. In this study, we compared the susceptibility of C3H/HeN mice to two highly virulent strains of *R. rickettsii*, one from the United States (Sheila Smith) and the other from Brazil (Taiaçu). Animals inoculated with the Taiaçu strain succumbed to infection earlier and exhibited severe histological lesions in both liver and spleen sooner than mice infected with the Sheila Smith strain. These differences in survival and signs of the disease are not related to a greater proliferation of the Taiaçu strain, as there were no significant differences in the rickettsial load in mice tissues inoculated with either strain. The present study is the first step to experimentally assess differences in fatality rates of RMSF in two different regions of the American continent.

## 1. Introduction

The genus *Rickettsia* (order Rickettsiales, family Rickettsiacea) contains 27 recognized species, among which at least 17 species are capable of causing diseases in humans [1]. *Rickettsia rickettsii* is the etiological agent of Rocky Mountain spotted fever (RMSF), the most lethal rickettsiosis that is transmitted to humans by ticks. The distribution of RMSF geographically overlaps with the areas where the tick species implicated as biological vectors are present. In North America, two *Dermacentor* species, *Dermacentor variabilis* and *Dermacentor andersoni*, are the main vectors of *R. rickettsii* [2]. In this region, *Amblyomma americanum* and *Rhipicephalus sanguineus* are also recognized as vectors [3,4]. In Central and South America, *R. rickettsii* is mainly transmitted by ticks in the *Amblyomma cajennense* species complex [5,6]. In Brazil, *Amblyomma sculptum*, one *A. cajennense* species complex member, and *Amblyomma aureolatum* are implicated as vectors [6].

The first records of RMSF date to 1873, with the description of a disease in the Western United States referred to as “spotted fever” or “black measles” (reviewed by [7]). Pioneering studies conducted in the 1900s concluded that *Dermacentor* spp. ticks were responsible for the transmission of the still unknown infectious agent to mammals, identified only few years later (reviewed by [1,8]). Subsequently, several cases of RMSF have been described in the Central to Southeastern United States as well as in Central and South America [7]. In Brazil, the first cases of RMSF were reported in the 1930s and referred to as “São Paulo exanthematic typhus” or “Minas Gerais exanthematic typhus.” Upon confirmation that the Brazilian disease shared the same etiology with the North American counterpart, the disease became known as Brazilian spotted fever (BSF) [9]. While few cases of BSF were recorded between the 1950s to the early 1980s, the number of cases has steadily increased since the end of the 1980s, demonstrating a rapid emergence of this disease in Brazil [6].

After transmission to a vertebrate host through the bite of an infected tick, *R. rickettsii* preferentially invades endothelial cells and then disseminates from the initial inoculation site, causing a systemic infection. Morbidity and mortality are associated with the systemic proliferation of the bacterium in the endothelium that irrigates multiple organs [10,11,12]. The onset of RMSF symptoms occurs between 2–14 days after transmission, beginning with high fever, severe headache, myalgia, anorexia, nausea, vomiting, abdominal pain and photophobia [8]. Moreover, most patients develop a maculopapular rash that results from the vasculitis caused by the bacterial proliferation within the endothelium. The rash commonly begins with small and pink macules, typically on the wrists, ankles and forearms, and evolves to maculopapules, quickly spreading to legs, buttocks, arms, axillae, trunk and neck [7,8,13]. Antibiotic-based treatment of RMSF is effective, but only if started in the early phase of the disease; however, clinical signs of infection are common to other infectious diseases, which frequently leads to an incorrect diagnosis with subsequent increase in lethality [8,14].

Over 4000 cases of RMSF are recorded every year in the United States [15]. On the other hand, only ~100 cases are confirmed annually in Brazil [16]. Nonetheless, while case fatality rates in the United States are low (~5–10%) [17,18], in Brazil, they oscillate around 40% and can surpass 70% in the state of Sao Paulo [19]. These distinct fatality rates of the disease in the United States and in Brazil suggest that differences in tick vectors, population susceptibility, and/or variation in virulence of the rickettsial strains exists. In addition, the early treatment with doxycycline, adopted in United States (as recommended by the Centers of Disease Control [20]), but not in Brazil, may also exert an important influence in the fatality rates. In the present study, we compared the susceptibility of C3H/HeN mice, which are known to be susceptible to *R. rickettsii* [21,22,23,24,25], to an intravenous infection with the strains Sheila Smith (American) and Taiaçu (Brazilian) of this bacterium.

## 2. Results

To compare the virulence of two different strains of *R. rickettsii*, Sheila Smith from the United States and Taiaçu from Brazil, we employed a murine model of infection, C3H/HeN. To eliminate variability associated with host susceptibility or vector capacity, sex- and age-matched animals were intravenously inoculated into the retroorbital sinus with the same dose of each strain and evaluated for clinical signs, survival, histopathology and bacterial load.

### 2.1. Clinical Signs and Survival Curve of C3H/HeN after Challenge with *R. rickettsii*

**Figure 1:**
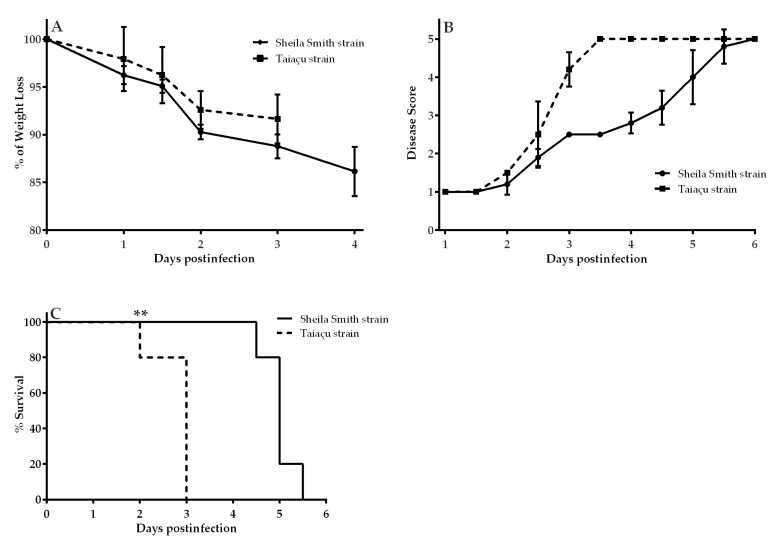
Monitoring of clinical signs and survival curve of C3H/HeN mice after challenge with two different strains of *R. rickettsii*. After challenge with Sheila Smith or Taiaçu strains, the mice were monitored twice daily for % of Weight Loss (**A**), Disease Score (**B**) and % of Survival (**C**). ** *p* < 0.05, Log-rank (Mantel-Cox) test.

In the first experiment, the progression of clinical signs of infection and survival of animals was evaluated (Figure 1). A similar percentage of body weight loss was recorded in the animals of both groups (Figure 1A). Observation of the clinical signs of infection showed that Taiaçu-infected mice reached the acute phase of disease (score 4) at 3 days postinfection (dpi), while Sheila Smith infected animals had a slower disease progression, reaching acute morbidity only 5 dpi (Figure 1B). Differences were also detected in the survival curve of animals inoculated with either the Sheila Smith or the Taiaçu strains (Figure 1C). Most of the Taiaçu-infected animals succumbed to the infection at 3 dpi. On the other hand, Sheila Smith infected animals survived for additional 2–2.5 days, reaching 100% of mortality at 5.5 dpi.

### 2.2. Dermination of *R. rickettsii* Load in C3H/HeN

**Figure 2:**
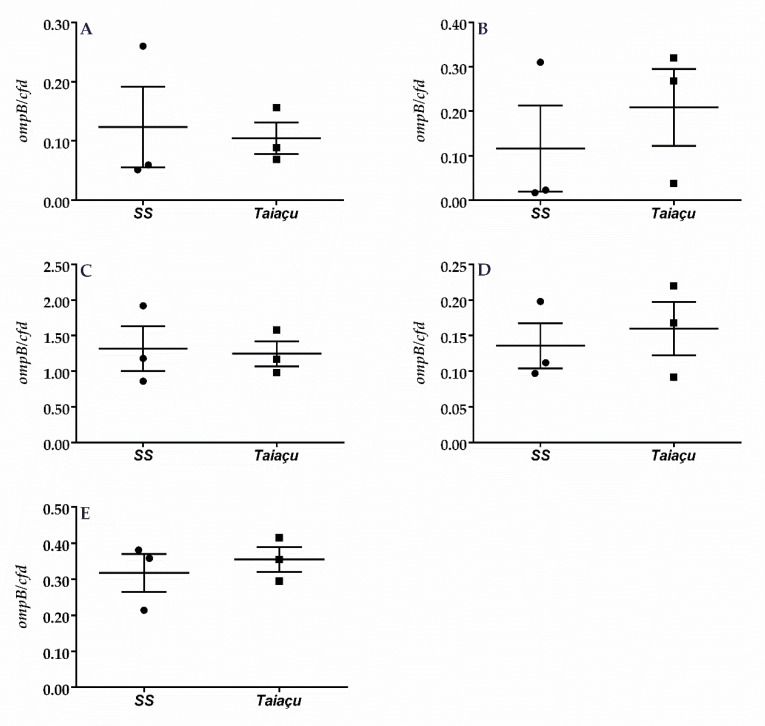
Determination of *R. rickettsii* load in C3H/HeN organs. The rickettsial load at 3 days postinfection in spleen (**A**), kidney (**B**), liver (**C**), heart (**D**) and lung (**E**) was determined by qPCR. Data represent the number of rickettsial *ompB* copies relative to mouse *cfd* copies. SS: Sheila Smith.

Having established apparent differences in virulence between each strain, we sought to determine the relationship between the bacterial load and mortality. To this end, we quantified *R. rickettsii* in the organs of mice inoculated with either Sheila Smith or Taiaçu strains, at the point where Taiaçu-infected mice were succumbing to the infection, which occurred at 3 dpi (Figure 2). Despite the differences in clinical signs and survival, significant differences were not detected in the rickettsial load in any organ of mice from both groups (Figure 2). However, a slightly higher but non-significant rickettsial load was detected in the kidney of mice infected with the Taiaçu strain (Figure 2B).

### 2.3. Histopathology and Immunohistochemistry Analyses of the Organs Infected with *R. rickettsii*

**Figure 3:**
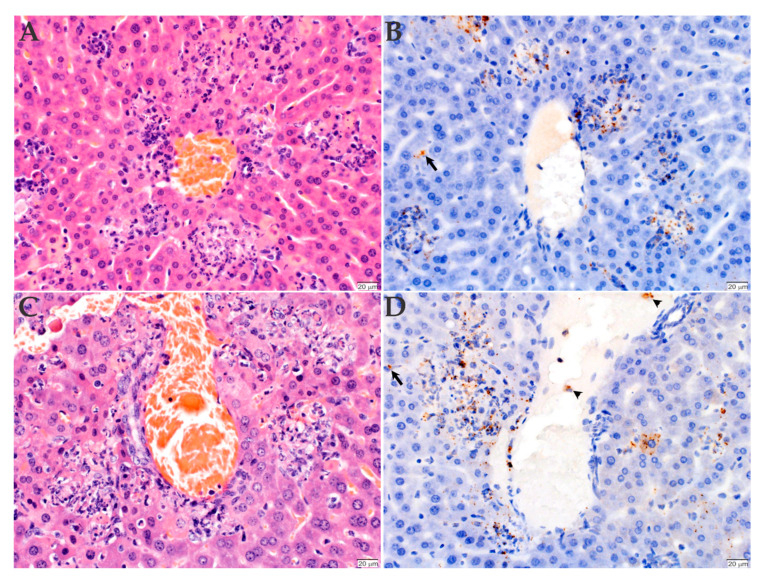
Histopathology and immunohistochemistry analyses of the liver of C3H/HeN mice inoculated with *R. rickettsii*. The liver of animals inoculated with either Sheila Smith (**A**,**B**) or Taiaçu (**C**,**D**) strains were stained with hematoxylin and eosin (**A**,**C**) or processed for anti-*R. rickettsii* immunohistochemistry (brown) with hematoxylin counter stain (**B**,**D**). Mice infected with both strains had acute multifocal to coalescing necrotizing hepatitis, with numerous intralesional degenerate neutrophils and macrophages (**A**,**C**). These lesions were associated with numerous intra- and extracellular rickettsial organisms within the macrophages and neutrophils in the necrotic foci (**B**,**D**), as well as in individual Kupffer cells (arrows) and occasional monocytes in circulation (arrowheads). (Scale bar = 20 µm).

**Figure 4:**
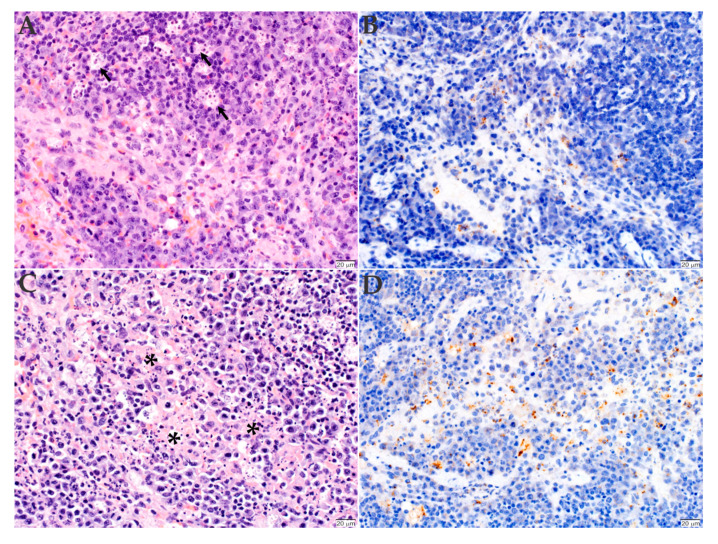
Histopathology and immunohistochemistry analyses of the spleen of C3H/HeN mice inoculated with *R. rickettsii*. The spleen of animals inoculated with either Sheila Smith (**A**,**B**) or Taiaçu (**C**,**D**) strain were stained with hematoxylin and eosin (**A**,**C**) or processed for anti-*R. rickettsii* immunohistochemistry (brown) with hematoxylin counter stain (**B**,**D**). The mice inoculated with the Sheila Smith strain of *R. rickettsii* had only individual tingible-body macrophages, with necrotic cell debris-laden cytoplasm (arrows, **A**) and rickettsiae (**B**). The splenitis of the mice inoculated with the Taiaçu strain was more severe, with more cells with nuclear debris (asterisks, **C**) and rickettsial organisms (**D**). (Scale bar = 20 µm).

To determine the lesions caused by the bacterial infection, the organs were also collected and processed for histopathology and immunohistochemistry analyses. All mice, independently of the inoculated *Rickettsia* strain, had acute multifocal to coalescing necrotizing hepatitis (Figure 3A,C) and splenitis (Figure 4A,C), with numerous intralesional degenerate neutrophils and macrophages. Immunohistochemistry of the liver revealed the presence of numerous positively labeled intra- and extracellular bacteria within the macrophages and neutrophils in the necrotizing lesions in this organ. A low number of bacteria were also evident in the cytoplasm of Kupffer cells (Figure 3B,D). The splenitis of the mice inoculated with the Sheila Smith strain was somewhat less severe than that of the mice inoculated with the Taiaçu strain of *R. rickettsii* (Figure 4A,C). Numerous bacteria are present in the cytoplasm of histiocytes in the red pulp and of a few tingible-body macrophages in the white pulp (Figure 4B,D).

**Figure 5:**
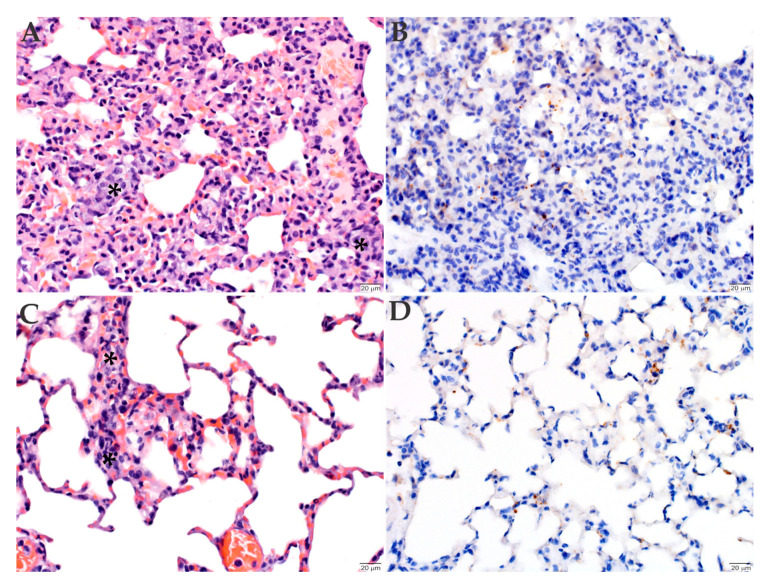
Histopathology and immunohistochemistry analyses of the lungs of C3H/HeN mice inoculated with *R. rickettsii*. The lungs of animals inoculated with either Sheila Smith (**A**,**B**) or Taiaçu (**C**,**D**) strain were stained with hematoxylin and eosin (**A**,**C**) or processed for anti-*R. rickettsii* immunohistochemistry (brown) with hematoxylin counter stain (**B**,**D**). The mice inoculated with the Sheila Smith strain had mild multifocal pneumonia, with occasional small intravascular aggregates of monocytes, individual karyorrhectic debris, and marginating leukocytes (asterisks, **A**). The mice inoculated with the Taiaçu strain showed increased number of circulating leukocytes in the lungs (asterisks, **C**). Individual cells within the alveolar septa have rickettsiae in their cytoplasm (**B**,**D**). (Scale bar = 20 µm).

The lungs of mice inoculated with the Sheila Smith strain had mild multifocal pneumonia, with scattered small intravascular aggregates of monocytes, individual karyorrhectic debris, and marginating leukocytes (Figure 5A). Conversely, the mice inoculated with the Taiaçu strain had an increased number of circulating leukocytes in the lungs (Figure 5C). The immunohistochemistry of the lung of mice inoculated with both strains indicated the presence of individual cells within the alveolar septa with rickettsiae in their cytoplasm (Figure 5B,D).

**Figure 6:**
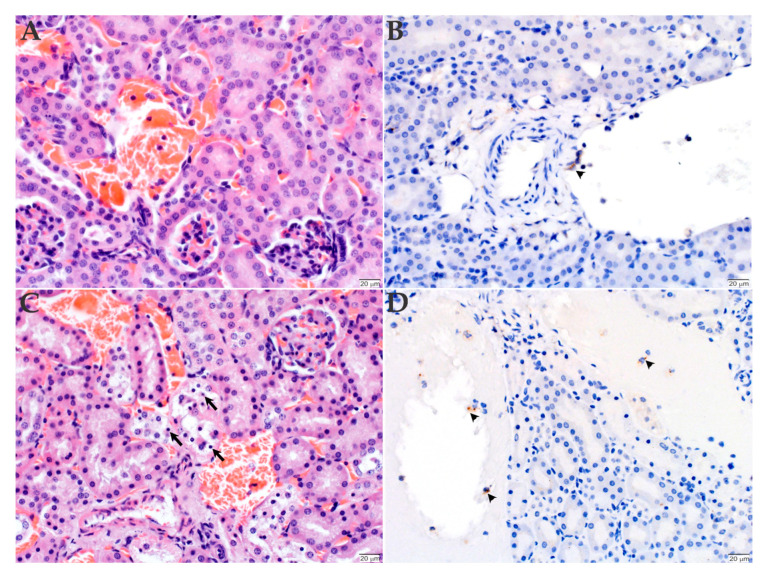
Histopathology and immunohistochemistry analyses of the kidneys of C3H/HeN mice inoculated with *R. rickettsii*. The kidneys of animals inoculated with either Sheila Smith (**A**,**B**) or Taiaçu (**C**,**D**) strain were stained with hematoxylin and eosin (**A**,**C**) or processed for anti-*R. rickettsii* immunohistochemistry (brown) with hematoxylin counter stain (**B**,**D**). The mice inoculated with the Sheila Smith strain were histologically unremarkable (**A**). The mice inoculated with the Taiaçu strain of *R. rickettsii* had individual renal cortical tubular epithelial cells with pyknotic nuclei and vacuolated cytoplasm, interpreted as cell degeneration and necrosis (arrows, **C**). Mice infected with both strains had individual circulating monocytes and endothelial cells lining blood vessels with immunolabeled microorganisms (arrowheads, **B**,**D**). (Scale bar = 20 µm).

The kidneys of the mice inoculated with the Sheila Smith strain were histologically unremarkable (Figure 6A), while the kidneys of Taiaçu had mild acute individual epithelial cell degeneration and necrosis in the distal tubules (Figure 6C). The immunohistochemistry of the kidney of mice inoculated with both strains revealed individual circulating monocytes and endothelial cells lining blood vessels with immunolabeled microorganisms (Figure 6B,D). No inflammation was evident in the kidneys, nor in the heart. Bacteria were also evident in the cytoplasm of individual endothelial cells lining the lumen and in monocytes circulating in vessels in all tissues, including the lung, kidney and heart (data not shown). In addition, no histological changes or immunohistochemical labeling were observed in the tissues of the control mouse (Supplementary Figure S1A–H).

## 3. Discussion

Although the fatality rate of RMSF in the United States is below 5%, in Brazil, it can surpass 70%. The distinct fatality rates suggest that differences in the virulence of American and Brazilian rickettsial strains may exist; however, part of this drastic difference could also be related to other factors, such as distinct numbers of recorded cases in these two countries. Recent studies have demonstrated a vaccine-mediated protection against *R. rickettsii* in C3H/HeN mice, which are susceptible to infection [21,22,23,24,25]. On the other hand, *R. rickettsii* failed to cause disseminated disease in C3H/HePas mice [26], even though this strain is closely related to C3H/HeN. Therefore, in the present study, we used C3H/HeN mice as a model to compare the infection with the strains Sheila Smith (American) and Taiaçu (Brazilian) of *R. rickettsii*.

Experimental animals were inoculated by the same route and with the same dose of each strain, avoiding variability associated with both host susceptibility or vector capacity. Infected animals were continuously evaluated for clinical signs, survival, bacterial load, and pathology. Animals infected with the Taiaçu strain reached the highest disease scores and succumbed to infection in a shorter time than those infected with the Sheila Smith strain. Of note, both morbidity and mortality timelines demonstrate that the animals did not succumb to the mouse toxic death phenomenon, which occurs around 18–24 h due to a massive simultaneous rickettsial penetration of the endothelium [27,28]. This decrease in survival time was not associated with a higher bacterial load; however, mice infected with Taiaçu exhibited severe histological lesions in both liver and spleen sooner than mice infected with the Sheila Smith strain. Interestingly, similar severe lesions were previously observed in C3H/HeN infected with the Sheila Smith strain 5 days after inoculation [21].

Previous studies have already reported differences in virulence of distinct strains of *R. rickettsii* after intradermal or intraperitoneal infection of guinea pigs [29,30,31]. For example, infection with strains of *R. rickettsii* obtained from patients and ticks from both Eastern and Western United States showed that western strains (Sheila Smith, Norgaardare and Sawtooth ♀ 2) are more virulent than eastern strains (Morgan and Simpson) [29]. In a more recent study, the Sheila Smith strain triggered a higher fever response in animals when compared to the Morgan and R strains [30]. Moreover, the strain HLP7421 caused only a limited fever response while the Iowa strain caused no response at all. Interestingly, Clark and collaborators [30] also evaluated the virulence of a Brazilian strain, named São Paulo, which caused a lower fever response in guinea pigs than the Sheila Smith strain. The current study has also demonstrated difference in virulence between the Sheila Smith and another Brazilian strain, Taiaçu, which was associated with a more rapid disease progression in mice. However, it is not possible to extrapolate overarching conclusions from these two studies as different methods were used, including the vertebrate model (guinea pig vs. mice), the Brazilian strains of *R. rickettsii* [(São Paulo isolated from *A. sculptum* (formerly named *A. cajennense*) vs. Taiaçu isolated from *A. aureolatum*)], the inoculation route (intradermal vs. retro-orbital), and the biological parameters that were evaluated (fever response vs. ruffled fur, hunched posture, shallow breathing, and immobility when touched), and associated with histopathology and immunohistochemistry analyses of mice organs. While it is not possible to conclude that the South American *R. rickettsii* strain is more virulent than the North American strain, the results of the current study support the premise that strain-dependent differences in virulence exist within both Brazilian and American *R. rickettsii* populations [17,32]. Indeed, it has been shown that there is a high polymorphism of rickettsial isolates in North American, where strains of different virulence degrees exist. Conversely, a lower polymorphism is observed in Central and South America isolates, where possibly only high virulent strains are found, correlating with the higher case fatality rates [17,18,33,34,35].

One hypothesis that could explain differences in strain-specific virulence is that virulence correlates with genomic differences between strains. Indeed, comparison of the genomes of two *R. rickettsii* strains, the virulent Sheila Smith [27] and the avirulent Iowa [36], showed that although the strains share a high degree of sequence identity, several deletions were detected in the avirulent strain [37]. Specifically, two deletions in the gene encoding the rickettsial outer membrane protein A (rOmpA) were observed in the genome of the Iowa strain, which results in the absence of this protein [37]. OmpA is involved in the attachment of the bacterium to the host cell and, therefore, is associated with virulence [38]. Interestingly, an *ompA* disrupted mutant of the Sheila Smith strain exhibited the same invasion and proliferation phenotype in Vero cells as the wild-type strain, suggesting the involvement of multiple proteins in the attachment of bacteria to host cells [39]. Additionally, the Iowa strain is also defective in processing of another important outer membrane protein, rOmpB [37,40]. rOmpB has also been associated with rickettsial virulence [41].

The small genome size of bacteria, including bacteria in the genus *Rickettsia*, has been associated with virulence, as higher rates of genome degradation were observed in the most virulent species [14,42]. Comparisons between *R. rickettsii* strains Sheila Smith and Iowa identified the exclusion of a large region (~10 kb) in the Sheila Smith genome, which was hypothesized to be an intermediate step in reduction of genes not essential for the survival of bacteria in both the vector or the host vertebrate [37]. Therefore, the sequencing of the genome of the Taiaçu strain and the comparative analysis with the genomes of other *R. rickettsii* strains is warranted and may identify molecular determinants responsible for its apparent higher degree of virulence. Importantly, Taiaçu and Sheila Smith strains were reported to respond differentially to environmental stimuli previously associated to the reactivation of rickettsial virulence [43,44,45,46]. While a temperature upshift and the acquisition of a blood meal by the tick vector upregulates the transcription of virulence genes by the Taiaçu strains, including Type IV secretion system components and OmpB [47,48], a temperature upshift or iron limitation exerted only a limited effect on the gene expression of the Sheila Smith strain [49]. These data reinforce that differences in the virulence degrees of these two strains exist. Therefore, a comparative analysis of the gene expression of these two strains in mice organs is warranted and might also help to understand the virulence factors involved in the differences in histopathology.

The immune responses of both the host and the tick vectors to distinct rickettsial strains may also exert an effect on the bacterial virulence [50]. Interestingly, the tick species that transmit *R. rickettsii* in Brazil present remarkable differences in susceptibility to infection, with *A. aureolatum* being more susceptible than *A. sculptum* [51]. This difference seems to be due to a distinct transcriptional response of immune factors in the tick midgut, which are mostly upregulated in *A. sculptum* and downregulated in *A. aureolatum* [52]. In addition, while the midgut of *A. sculptum* presents an incipient microbiota, *A. aureolatum* exhibits a prominent microbiota, composed mostly by bacteria of the genus *Francisella*. Therefore, it is possible that *Francisella* might desensitize, instead of prime, the immune system of *A. aureolatum* [53]. The susceptibility of *A. sculptum* to infection is also somewhat higher in ticks exposed to autochthone than non-autochthone *R. rickettsii* [54,55]. Together, these data suggest that the complex interaction between ticks and *R. rickettsii* culminates in different outcomes of infection, which may also play a role in the differences in RMSF fatality rates. Additional studies are required to elucidate the rickettsial and murine molecular factors as well as to determine the influence of the tick vectors on the pathogenic nature of *R. rickettsii*.

## 4. Materials and Methods

### 4.1. *R. rickettsii* Cultivation and Purification

Two different strains of *R. rickettsii* were used: Sheila Smith and Taiaçu. The Sheila Smith strain was isolated from a human in Western Montana, United States, in 1946 [27], while the Taiaçu strain was isolated from a naturally infected *A. aureolatum* tick from the rural area of Taiaçupeba, Mogi das Cruzes County, state of São Paulo, Brazil [56]. The Sheila Smith strain was submitted to three passages in Vero cells since its egg yolk sac passage. The time course of morbidity and mortality was consistent with previous observations with intravenous inoculation of Sheila Smith strain with the same passage history [21,57]. The Taiaçu strain was submitted to twenty-two passages in Vero cells since the last guinea pig passage. To obtain purified rickettsiae of both strains, infected Vero cells were lysed by repeated passage through a needle, centrifuged over a 20% sucrose cushion (16,000× *g*, 4 °C, 30 min) and stored at −80 °C in SPG buffer (218 mM sucrose, 3.8 mM KH_2_PO_4_, 7.2 mM K_2_HPO_4_, 4.9 mM L-glutamate, pH 7.2), as previously described [58]. The bacteria were quantified by endpoint dilution assay as described Reed and Muench (1938) [59]. Briefly, bacteria were serially diluted (10× fold dilutions) and 10 μL of each dilution were added to 8 or 16 wells of confluent Vero cells in 96 well plates. After inducing contact between the bacteria and the host cell by centrifugation at 500× *g*, the plate was incubated in DMEM with 5% fetal bovine serum (FBS), non-essential amino acids, and sodium pyruvate at 34 °C 5% CO_2_ for 6 days. The cells were fixed using 4% paraformaldehyde solution, permeabilized with 0.1% Triton X-100, blocked with 2% bovine serum albumin, and stained with the rabbit anti-*Rickettsia* antibody Rc-PFA and Alexafluor488 conjugated anti-Rabbit IgG. The presence of bacteria in each well was determined by fluorescence microscopy using common green fluorescence protein filters at 100× total magnification. The 50% tissue culture infective dose (TCID_50_) was determined as previously described, whereby 1 TCID_50_ mL^−1^ = 0.7 infectious units (IU) mL^−1^ [59].

### 4.2. Murine Infections

Mice (*Mus musculus*), 7–8-week-old male C3H/HeN, were obtained from Charles River Laboratories. This mice strain was used as it is susceptible to *R. rickettsii* Sheila Smith infection [21,22,23,24,25]. The intravenous infection route mimics the natural hematophagous arthropod transmission into the bloodstream and results in vascular infection. All procedures involving animals were carried out in accordance with permission from the Institutional Animal Care and Use Committee at the Louisiana State University (protocol 15–115) (IACUC-LSU).

All mice groups were age- and sex-matched and randomly distributed into experimental groups. For mouse examination, cages were open in a random order whereby the observant did not know the identity of the treatment prior to scoring. To describe the clinical progression of disease, mice were observed for clinical signs of infection twice per day and were weighed every 24 h. Each animal was scored for clinical signs of infection based on the following scale: (1) Infected but with no observable signs of infection, (2) Ruffled fur, decreased activity, slightly hunched posture, 5–10% weight loss, (3) Ruffled fur, hunched posture, difficulty maintaining posture, reduced prostration, shallowed respiration, 10–15% weight loss, (4) >15% weight loss, limited motility or prostration. (5) Deceased. All animals that scored >3 were removed from the study and considered as succumbing to infection (represented with a score 5).

A pilot experiment consisted of 5 animals in each group. These numbers were chosen to determine the time course of morbidity and mortality for both strains. The animals were anesthetized intraperitoneally with 100 mg kg^−1^ ketamine and 4 mg kg^−1^ xylazine and challenged with 1 lethal dose (1LD = 1.0 × 10^7^ infectious unit) of *R. rickettsii* diluted in 0.1 mL of PBS buffer by intravenous retro-orbital injection. To further analyze the bacterial dissemination and histopathology, we infected 3 additional mice with each *R. rickettsii* strain. These mice were euthanized at 3 days postinfection, when the Taiaçu-infected animals were succumbing to infection. Approximately 10 mg of spleen, kidney, liver, heart and lung were collected and kept at −80 °C until processing to determine the rickettsial load by real-time quantitative PCR (qPCR). A portion of these same tissues were also fixed in formalin for histological and immunohistochemical analyses. Tissues samples from naïve mice were used as a control.

### 4.3. DNA Extraction and Determination of *R. rickettsii* Load

Genomic DNA (gDNA) was extracted from mouse organs using the DNeasy^®^ Blood & Tissue Kit (Qiagen, Hilden, North Rhine-Westphalia, Germany) as described by [60]. gDNA was then used as template in qPCR using specific primers for a single copy gene of either *R. rickettsii* (*ompB*) [61] or mouse (*cfd*: Complement Factor D) [60]. The qPCR reactions were performed in a LightCycler 480 system II (Roche, Basel, Switzerland) and the LightCycler 480 software, as described by [60]. The quantitation cycle (Cq) of each sample was compared with the Cq of standard curves constructed with serial dilutions of plasmids containing a fragment of either *ompB* or *cfd* and used to calculate the relative number of rickettsiae to mouse *cfd* copies.

### 4.4. Histopathology and Immunohistochemistry

Selected mouse organs (liver, spleen, kidney, lung and heart) were fixed in 10% buffered formalin, embedded in paraffin, sectioned, and stained with hematoxylin and eosin (H&E). Additional serial sections of all tissues were assessed by indirect immunohistochemistry (IHC) for the presence of *Rickettsia* using a rabbit anti-RcPFA polyclonal antibody (1:500 dilution) that recognizes *R. rickettsii* [58] for 30 min using the antigen retrieval and polymer refine detection protocols of the BOND-MAX (Leica Biosystems, Wetzlar, Hesse, Germany) automated immunohistochemistry stainer utilized for this procedure.

### 4.5. Statistical Analysis

The statistical analysis of rickettsial load in mouse tissues inoculated with either the Sheila Smith or Taiaçu strains were calculated using Student’s t test. For the survival curve, the Log-rank (Mantel–Cox) test was used. The analyses were performed in GraphPad Prism version 7.0 for Windows (GraphPad Software, San Diego, California, USA) and a *p* value ≤ 0.05 was considered statistically significant.

## Supplementary Materials

The following are available online at https://www.mdpi.com/2076-0817/9/9/744/s1, Figure S1: Histopathology and immunohistochemistry analyses of the organs of uninfected C3H/HeN mice. As a control, the liver (**A**,**B**), spleen (**C**,**D**), lungs (**E**,**F**) and kidneys (**G**,**H**) of uninfected mice were stained with hematoxylin and eosin (**A**,**C**,**E**,**G**) or processed for anti-*R. rickettsii* immunohistochemistry (brown) with hematoxylin counter stain (**B**,**D**,**F**,**H**). No histological changes or immunohistochemical labeling were observed in the organs of control mouse. (Scale bar = 20 µm).
