# Supplementary material for: Comparative Analysis of Infection by Rickettsia rickettsii Sheila Smith and Taiaçu Strains in a Murine Model
Source: Pathogens. 2020 Sep 10;9(9):744. doi: 10.3390/pathogens9090744 (PMC7557639; doi:10.3390/pathogens9090744)
Supplement: Supplementary file 1 [file pathogens-09-00744-s001.pdf]

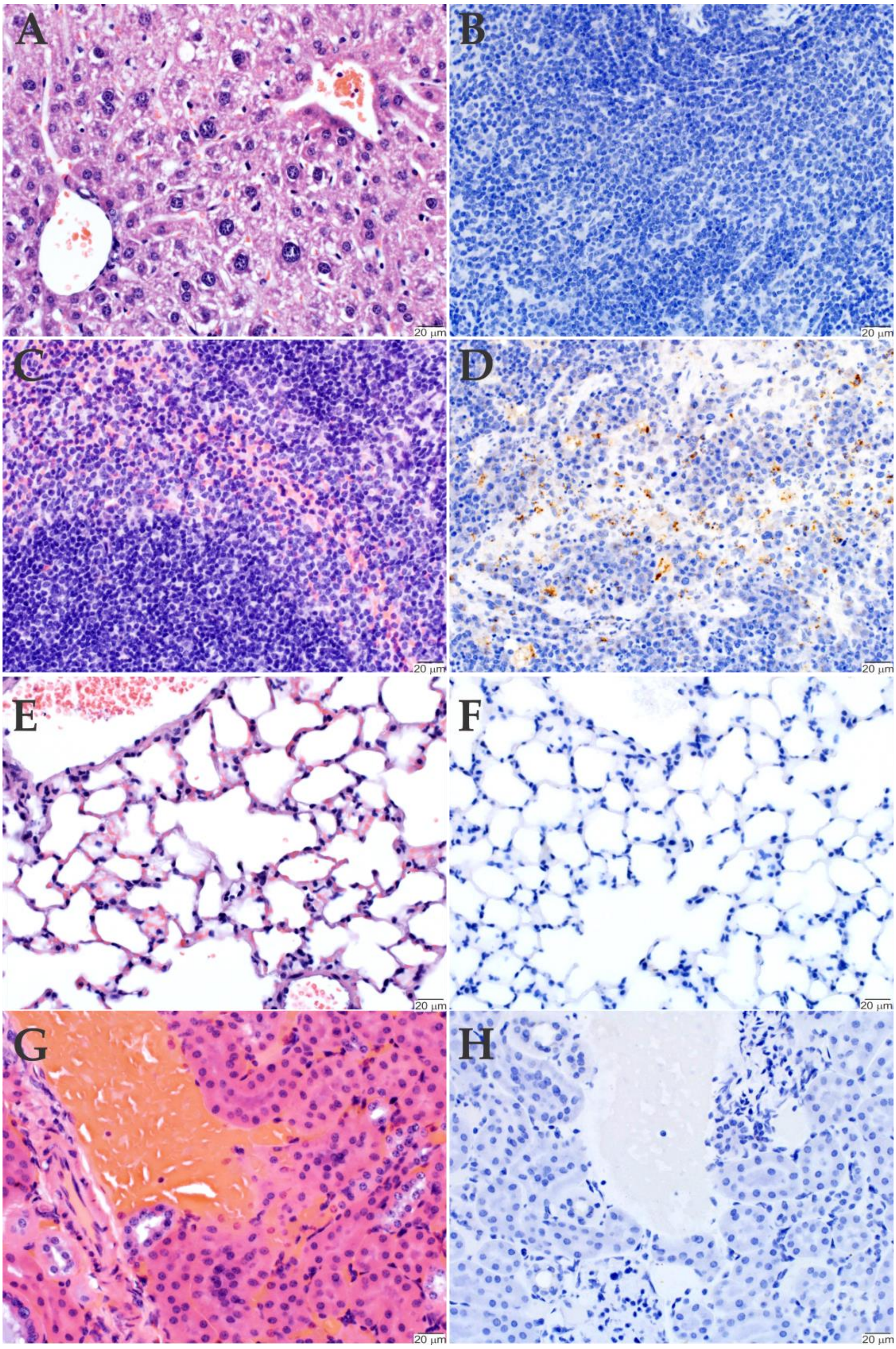

Figure S1. Histopathology and immunohistochemistry analyses of the organs of uninfected C3H/HeN mice. As a control, the liver (**A**,**B**), spleen (**C**,**D**), lungs (**E**,**F**) and kidneys (**G**,**H**) of uninfected mice were stained with hematoxylin and eosin (**A**,**C**,**E**,**G**) or processed for anti-*R. rickettsii* immunohistochemistry (brown) with hematoxylin counter stain (**B**,**D**,**F**,**H**). No histological changes or immunohistochemical labeling were observed in the organs of control mouse. (Scale bar = 20 μm).
